# A Low-Cost, Low-Tech Virtual Mass Casualty Training Simulation for Undergraduate Medical Education

**DOI:** 10.7759/cureus.69603

**Published:** 2024-09-17

**Authors:** Samuel Kolb, Puja Patel, Vishnu Mudrakola, Dhimitri A Nikolla, Amanda Lee, Dusty Barbour, Kaitlin M Bowers

**Affiliations:** 1 Department of Emergency Medicine, Jerry M. Wallace School of Osteopathic Medicine, Campbell University, Lillington, USA; 2 Department of Emergency Medicine, Summa Health, Akron, USA; 3 Department of Internal Medicine/Emergency Medicine, Lake Erie College of Osteopathic Medicine, Erie, USA; 4 Department of Emergency Medicine, Allegheny Health Network, Erie, USA

**Keywords:** disaster, disaster preparedness, disaster response and preparedness, field triage, mass casualty incident, multi-victim, simulation, triage, undergraduate medical education, virtual training models

## Abstract

Background: Mass casualty incident (MCI) training effectively increases trainees' knowledge and confidence when implemented in a live, in-person setting. In-person MCI training is resource-intensive, but virtual MCI training models are an alternative with similar effectiveness at a lesser cost. However, most of these validated virtual options are based on high-tech virtual reality (VR) programs. We designed and implemented a low-tech, low-cost virtual MCI training model for third-year medical students, using Google Jamboard^TM^ (Google, Mountain View, CA) and Zoom^TM^ (Zoom Video Communications, Inc., San Jose, CA) as the primary technological platforms.

Methods: Learners were instructed on the adult simple triage and rapid treatment (START) and the pediatric JumpSTART triage algorithms over Zoom^TM^. In small groups, students used a gameboard on Google Jamboard^TM^ to simulate a scene at an MCI where they were tasked with triaging 25 patients in 30 minutes, followed by a debriefing session. Students were surveyed on their perceived understanding of the triage algorithms and confidence before and after the event using a 5-point Likert scale (poor, fair, good, very good, and excellent). Pre- and post-event scores were compared using paired, two-sample, and two-tailed t-tests. We considered a p value of <0.01 significant to correct for multiplicity using the Bonferroni method.

Results: Learners reported an increased understanding of the triage algorithms (adult and pediatric), scene setup, and understanding of emergency medical service training/transportation, as well as increased confidence in participating in an MCI (all p < 0.001).

Conclusions: Virtual MCI training can be used as an alternative or supplement to in-person MCI training. Low-tech virtual MCI training models can increase the accessibility of these valuable training activities without sacrificing the quality of learning. Areas for further investigation include low-tech virtual MCI training models' ability to effectively recreate situational and environmental distractions and other challenges better simulated by in-person and high-tech VR training events.

## Introduction

Mass casualty incident (MCI) triage training has been adapted across multiple hospital systems to allow hospital staff to familiarize themselves with algorithms and move efficiently. A review of 20 international case reports documenting natural disasters and their subsequent medical responses concluded that the primary challenges when managing mass casualty or disaster events were related to the provider's ability to manage uncertainty and surprising situations [1]. Implementing structured processes to respond to these often unforeseen scenarios is paramount. Ideally, it should be tailored to the forms of MCI most commonly seen in a particular geographical region or setting [2]. MCI training is effective in its traditional format, exposing trainees to simulated, in-person MCI events, such as natural disasters, mass transportation accidents, and acts of terrorism [3].

This traditional implementation strategy for MCI training, involving live actors, moulage, trainers, and other volunteers, has been demonstrated to increase learners' knowledge, confidence, and performance in assigning triage designations based on pre- and post-simulation data [3,4]. In-person training has also been shown to elicit sympathetic nervous system responses from participants, approximating the same sympathetic response participants would be expected to experience during a true MCI [5].

Cost is a major barrier to implementing traditional in-person MCI training. One Canadian model estimated that the development and implementation of their in-person model required 200 person-hours for curriculum development, 68 person-hours for actors, and six hours of pre- and post-training didactic activities to run a two-hour MCI drill for medical students. The estimated total cost for the computer-aided design was 22,000 (185 USD/student) [6]. In-person MCI training also has limited generalizability. For example, many preexisting MCI training programs are based on the availability of first-world healthcare resources and do not apply to resource-limited areas [7]. For this reason, logistical training has been identified as an educational point of emphasis over medical knowledge and skills [7].

Virtual implementation of MCI training offers an alternative. Multiple studies have shown that training healthcare teams in online, virtual environments with dynamic virtual patients is an effective training method for future management of MCIs [8-10]. While realism is an obvious concern with virtual MCI training, multiple studies have shown that a high-tech virtual reality (VR) platform can create the same high-pressure environment for learners as in-person training, measured by physical signs of sympathetic activation in study participants [5]. This effect has been demonstrated in medical students, residents, and attendings [11].

The foremost benefit of VR MCI training is its cost-effectiveness relative to traditional training. An Australian study demonstrated a 13-fold decrease in cost by comparing an in-person MCI training method for paramedics with a new VR-based method while maintaining a nearly identical degree of effectiveness [12]. In these ways, virtual systems may be an adequate replacement for expensive conventional training methods and a safe and efficient platform for research on current triage protocols [13]. However, multiple studies comparing the efficacy of VR to traditional in-person MCI training have concluded that virtual training platforms should not wholly overtake traditional training environments but rather serve as an adjunct to current training methods [14-16].

Most emerging research in the MCI training space involves validating the effectiveness of high-tech VR-based models, which require significant technological capability [17-21]. We aimed to examine pre- and post-event understanding of and confidence with MCI triage using a low-tech, virtual MCI training model among third-year medical students from a United States medical school before their clinical rotations.

## Materials and methods

**Figure 1:**
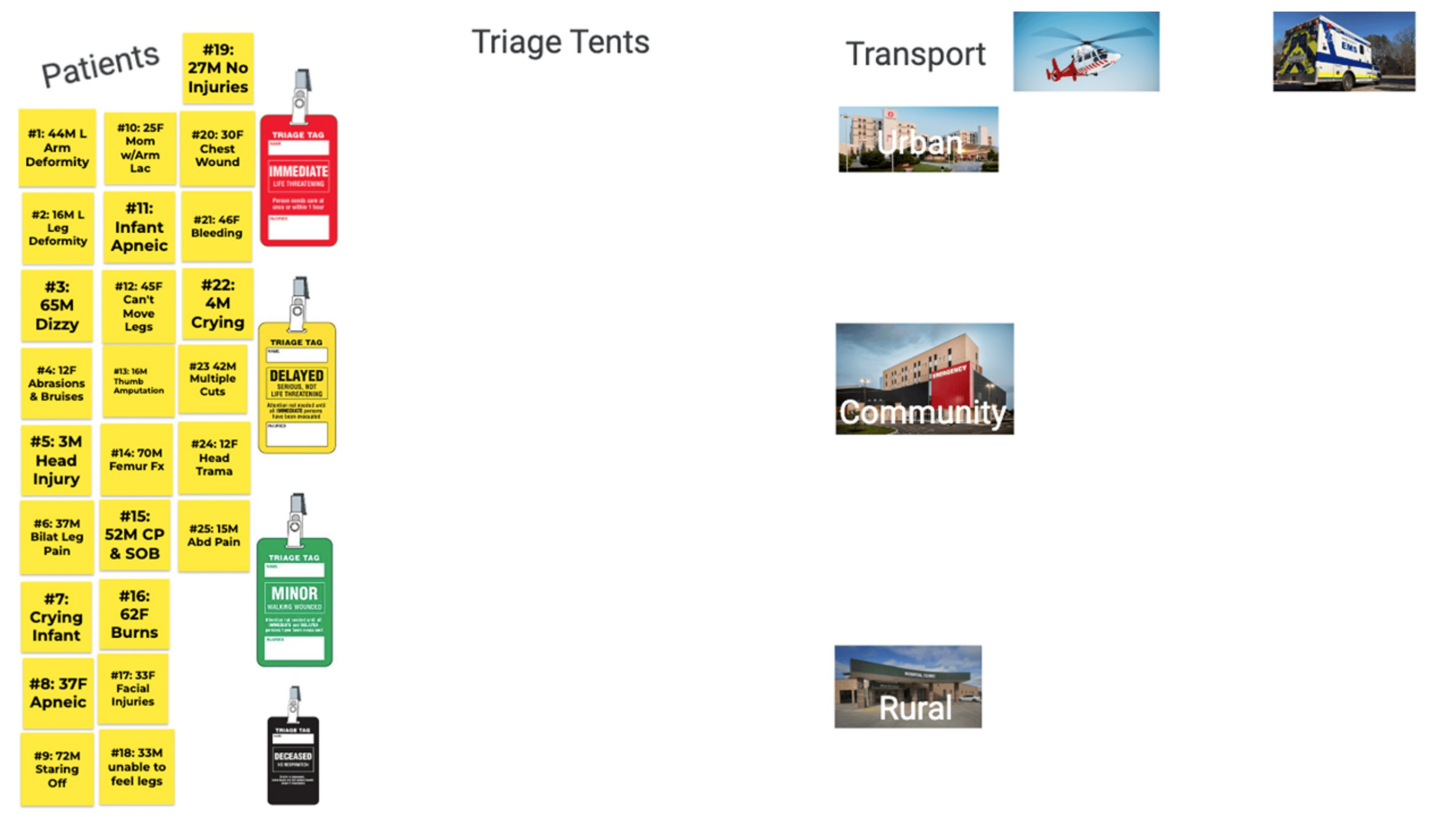
Google Jamboard at the beginning of the activity CP: chest pain; SOB: shortness of breath

The targeted learners were third-year medical students during their required simulation medicine rotation before starting clinical rotations. Students were given reading material before the activity to familiarize themselves with the triage algorithms [22]. The activity was performed using videoconferencing via Zoom^TM^ (Zoom Video Communications, Inc., San Jose, CA) and a digital whiteboard application through Google called Jamboard^TM ^(Google, Mountain View, CA). The event began with a brief lecture by a board-certified emergency physician introducing MCIs and teaching the adult simple triage and rapid treatment (START) and the pediatric JumpSTART triage algorithms. The lecture material aligned with the required reading [22]. Students were then broken into groups of five and instructed to assign roles (incident commander, triage supervisor, red tent supervisor, yellow tent supervisor, and green tent supervisor). Each breakout room was given a separate Google Jamboard^TM^ to screen share (Figure 1) with 25 game pieces, each representing a patient.

**Figure 2:**
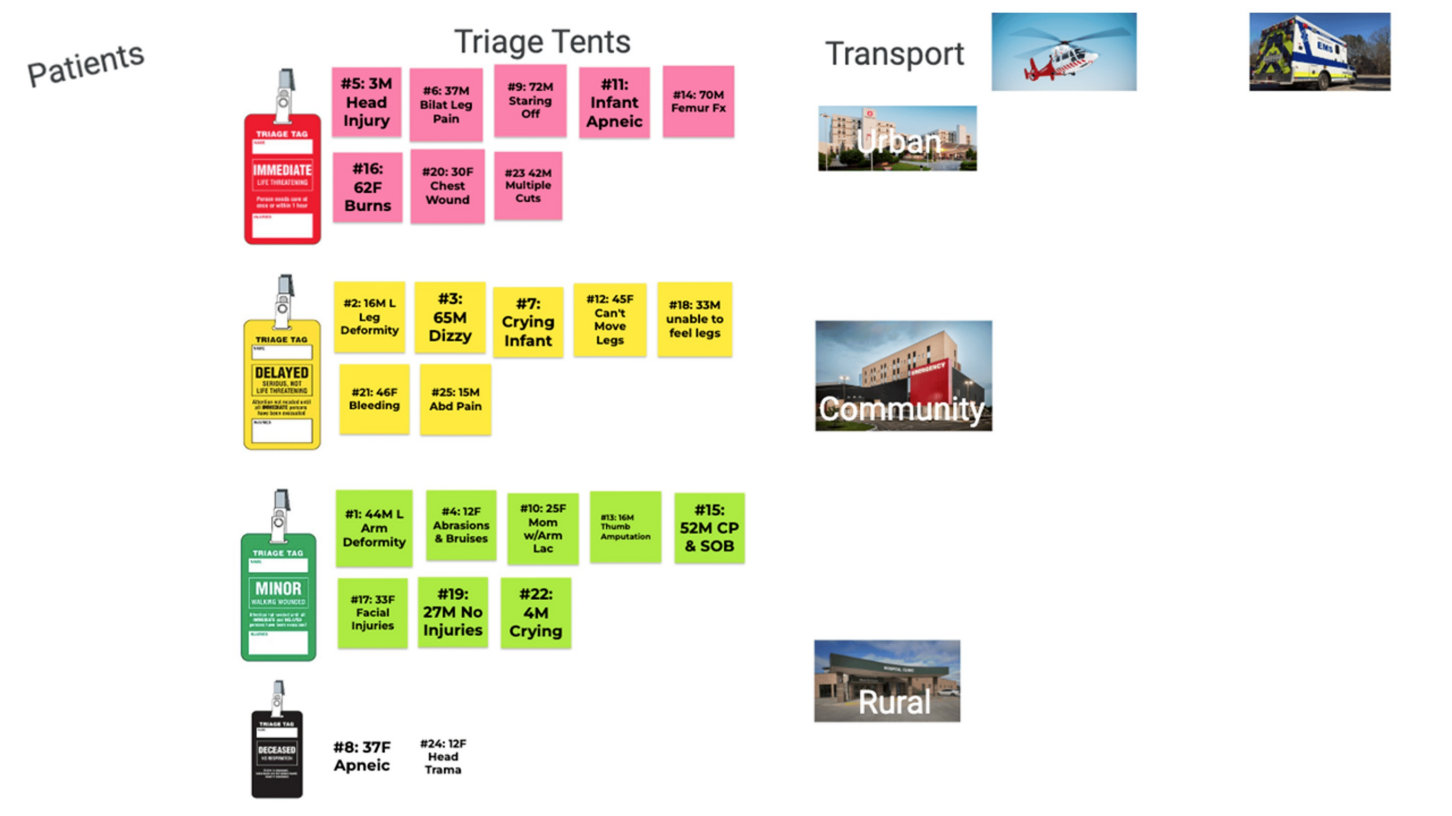
Google Jamboard after completing part one of the virtual MCI activity CP: chest pain; SOB: shortness of breath; MCI: mass casualty incident

A board-certified emergency physician created the simulated patients, and they included various medical and trauma complaints with varying severity of illness (i.e., red, yellow, green, and black tags). Facilitators were assigned to each room and given information about each patient's status to read when prompted by the students. These facilitators read prescripted assessments for each patient when prompted by the students and could not provide students with any additional information or guidance. Part one of the virtual MCI drill required the students to triage 25 victims on the Google Jamboard^TM^ in 30 minutes. Students moved the patients on the Jamboard^TM^ in real time so that the entire group could visualize. Any groups that finished with time remaining were asked to begin triaging/prioritizing patients within each color designation (Figure 2). When five minutes remained in the activity, announcements were sent to the breakout rooms stating that emergency vehicles (air and ground) had arrived to begin transporting patients to the hospital. Students were asked to determine in order which patients they would transport first.

**Figure 3:**
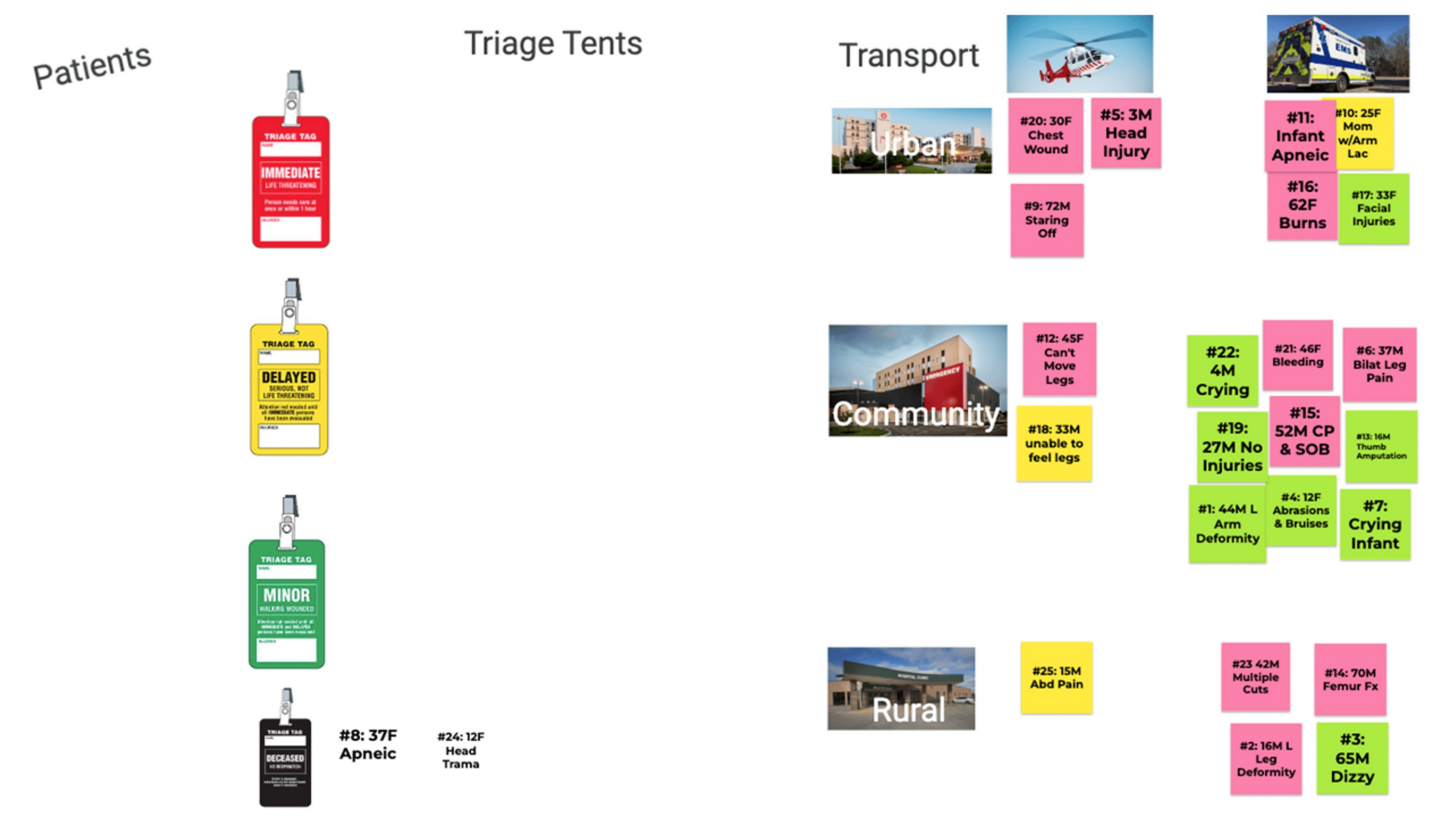
Google Jamboard after completing part two of the virtual MCI activity CP: chest pain; SOB: shortness of breath; MCI: mass casualty incident

After 30 minutes had passed, students returned to the main room for a debriefing. The debriefing was student-led but facilitated by a board-certified emergency physician to answer questions. Following the debriefing, students were given information on part two of the exercise, which required them to reassess all of their patients, re-triage as needed, and transport all remaining patients in 30 minutes. During this portion of the exercise, students were given information about three local hospitals (a rural, community, and tertiary care center) and the resources (specialists, emergency department beds, and number of operating rooms) each had. Students were required to specify which hospital patients were being transported to as transport vehicles arrived. A faculty member was again present in the breakout room with updates on each patient's condition to read when prompted by the students. Figure 3 shows one group's Google Jamboard^TM^ after completing part two of the exercise.

Part two concluded when all 25 patients had been transported or 30 minutes had elapsed. Students again returned to the main room for a debriefing. After the activity, students were asked to complete an optional survey (see the Appendices). The post-event survey consisted of five questions asking learners to rate their understanding of the triage algorithms reviewed, scene setup, and emergency medical service (EMS) transport types, as well as their overall confidence in participating in an MCI. Students rated their confidence before and after the activity on a Likert scale: 1: poor, 2: fair, 3: good, 4: very good, and 5: excellent. Prior EMS and medical experience were also question items. Only complete surveys were included in the analysis.

Parametric tests, such as the t-test, have performed well with Likert scale data despite concerns that the data are ordinal and not normally distributed [23]. Therefore, we used the paired, two-sample, two-tailed t-tests to compare group means for each survey question. We used the Bonferroni method to correct for multiplicity; therefore, we considered a p value of <0.01 significant (new alpha = original alpha / number of comparisons = 0.05 / 5 = 0.01). The Campbell University Institutional Review Board reviewed protocol #890 and determined that the study does not constitute human subjects research as defined by 45 CFR 46.102(e).

## Results

Of the 142 participants who took the survey, we excluded eight surveys for missing data and analyzed 134 complete surveys. Understanding of both the adult and pediatric triage algorithms increased upon completion of the virtual MCI activity (p < 0.001) (Table 1). Most learners ranked their post-event understanding as "very good" or "excellent," with 63% for adults and 58% for the pediatric triage algorithms, respectively. Further, there were zero responses ranking confidence as poor post-event.

**Table 1:** Post-MCI activity survey results MCI: mass casualty incident; SD: standard deviation; CI: confidence interval; DF: degrees of freedom; EMS: emergency medical services; MCI: mass casualty incident

Category	Before MCI, mean (SD)	After MCI, mean (SD)	Mean difference (95% CI)	DF	t-statistic	p value
Adult triage algorithm	2.31 (1.28)	3.82 (0.84)	1.51 (1.32-1.71)	133	15.54	<0.001
Pediatric triage algorithm	2.22 (1.26)	3.75 (0.87)	1.53 (1.33-1.72)	133	15.51	<0.001
Scene setup	2.48 (1.19)	3.90 (0.88)	1.42 (1.24-1.59)	133	16.08	<0.001
EMS training	2.71 (1.29)	3.94 (0.88)	1.23 (1.03-1.43)	133	12.06	<0.001
MCI confidence	2.41 (1.28)	3.75 (0.93)	1.34 (1.13-1.55)	133	12.61	<0.001

There was also a significant increase in learner understanding of scene setup (p < 0.001). The participants' overall confidence in their ability to take part in an MCI also showed a significant increase (p < 0.001), and the majority ranked their confidence post-event as "very good" or "excellent" (59%). The participants' understanding of EMS training also significantly increased (p < 0.001). Interestingly, 66 students (49%) had prior EMS and/or medical experience. Furthermore, 35 students (26%) participated in a prior MCI exercise.

## Discussion

Our analysis examined a low-tech MCI training model designed for third-year US-based medical students, utilizing Zoom^TM^ and Google Jamboard^TM^. Each technology involved is low-tech and low-cost and does not demand advanced computers or technological savvy to operate effectively. Despite the low-tech training environment and 30% of the medical students in this study having participated in a prior MCI exercise, participants indicated an increased understanding of adult and pediatric triage algorithms, scene setup, EMS resources, and confidence to effectively navigate a real-life MCI based on survey data before and after the training. These results were statistically significant despite correction for multiplicity.

The results of this study support the idea that virtual MCI triage training can increase understanding and confidence after participation, as previously outlined by multiple prior studies [8,9,12,14,15,21]. It also suggests that virtual MCI training may be valuable, even for learners with prior EMS training and MCI exercise experience. Therefore, low-tech models have the potential to increase exposure to MCI training earlier and more frequently throughout the formal education of physicians and other medical professionals. However, additional innovation and research are needed to improve and measure the efficacy of low-tech, virtual MCI training. For example, it is unknown if low-tech, virtual MCI training would be as effective among medical students who have completed some or most of their clinical rotations. Additionally, it is unknown how well low-tech virtual MCI training models recreate situational and environmental distractions compared to high-tech VR and in-person trainings.

Our low-tech, virtual MCI training model had several limitations, including its inability to simulate the variety of situational distractions and challenges that in-person and high-tech VR training models can. Examples of this include emotionally distressed or uncooperative patients, impaired communication due to loud noises and commotion, and the extra time necessary to navigate a large physically, often spread out MCI scene. Participants' sympathetic nervous system activity, as an approximation of induced stress, was not examined in this study, as has been in other virtual MCI formats [5,11]. Developing methods to incorporate a stress-inducing factor into the exercise warrants further study. Similarly, we could not simulate the various procedures and tasks expected of medical personnel at an MCI scene, such as applying direct pressure to stop bleeding, applying tourniquets, airway repositioning, carrying or moving patients, and physical assessments of patients. Additional limitations include recall and response biases since participants rated their pre-event understanding and confidence levels on a post-event survey.

## Conclusions

Our low-tech, virtual MCI training model improved participants' understanding of popular triage algorithms, and learners' confidence grew in efficiently triaging and allocating available resources. Performing MCI training virtually is a cost-effective method to provide an introduction to mass casualty events. By leveraging a low-tech format, virtual MCI training models can be more accessible. While the virtual format allows for a controlled, safe environment for students to wrestle with the newly presented algorithms, there remains a question as to whether this low-tech scenario successfully reproduces the same quality and intensity of distractions proven to be well-simulated by in-person and high-tech VR training modalities. However, based on this retrospective survey, our low-tech training option may be a low-cost, alternative or potential adjunct to in-person training, as many medical students are likely to be exposed to more robust in-person training later in their careers.

## Figures and Tables

**Figure 4:**
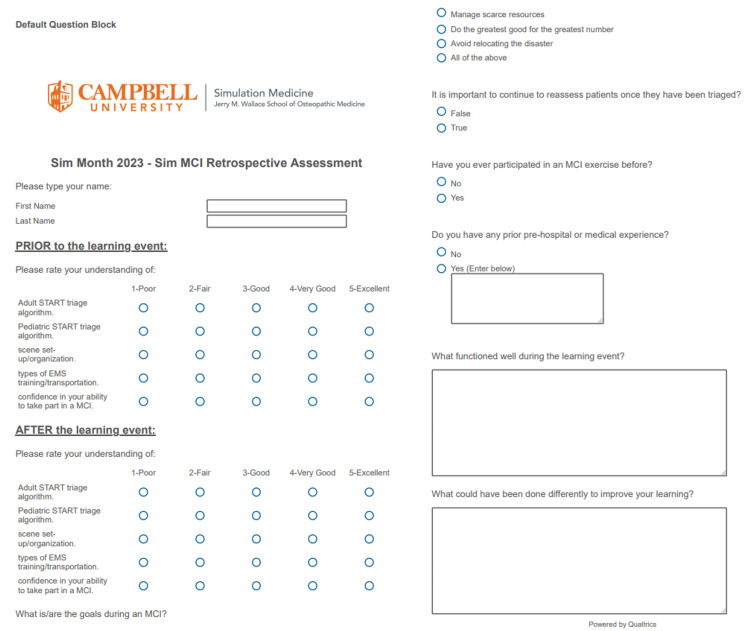
Post-event survey MCI: mass casualty incident; START: simple triage and rapid treatment; EMS: emergency medical service

## References

[1] Hugelius K, Becker J, Adolfsson A (2020). Five challenges when managing mass casualty or disaster situations: a review study. Int J Environ Res Public Health.

[2] Bazyar J, Farrokhi M, Khankeh H (2019). Triage systems in mass casualty incidents and disasters: a review study with a worldwide approach. Open Access Maced J Med Sci.

[3] Yu E, Coffey C (2024). Prehospital mass casualty incident triage simulation builds knowledge and confidence in medical trainees. AEM Educ Train.

[4] Gue S, Cohen S, Tassone M (2023). Disaster day: a simulation-based competition for educating emergency medicine residents and medical students on disaster medicine. Int J Emerg Med.

[5] Tovar MA, Zebley JA, Higgins M (2023). Exposure to a virtual reality mass-casualty simulation elicits a differential sympathetic response in medical trainees and attending physicians. Prehosp Disaster Med.

[6] Eastwood KW, Harris A, Armstrong JB (2023). A disaster medicine course for Canadian medical students: first implementation of a large-scale mass-casualty simulation. Can J Emerg Med.

[7] Leow JJ, Brundage SI, Kushner AL (2012). Mass casualty incident training in a resource-limited environment. Br J Surg.

[8] Heinrichs WL, Youngblood P, Harter P, Kusumoto L, Dev P (2010). Training healthcare personnel for mass-casualty incidents in a virtual emergency department: VED II. Prehosp Disaster Med.

[9] Andreatta PB, Maslowski E, Petty S (2010). Virtual reality triage training provides a viable solution for disaster-preparedness. Acad Emerg Med.

[10] Brinley Rajagopal A, Jasperse N, Boysen Osborn M (2020). Simulated mass casualty incident triage exercise for training medical personnel. J Educ Teach Emerg Med.

[11] Tovar M, Zebley J, Zwemer C (2023). Assessing the sympathetic response of medical doctors and trainees when exposed to a virtual realty mass casualty incident simulation. Prehosp Disaster Med.

[12] Mills B, Dykstra P, Hansen S (2020). Virtual reality triage training can provide comparable simulation efficacy for paramedicine students compared to live simulation-based scenarios. Prehosp Emerg Care.

[13] Kman NE, Price A, Berezina-Blackburn V (2023). First responder virtual reality simulator to train and assess emergency personnel for mass casualty response. J Am Coll Emerg Physicians Open.

[14] Heldring S, Jirwe M, Wihlborg J, Berg L, Lindström V (2024). Using high-fidelity virtual reality for mass-casualty incident training by first responders - a systematic review of the literature. Prehosp Disaster Med.

[15] Pucher PH, Batrick N, Taylor D, Chaudery M, Cohen D, Darzi A (2014). Virtual-world hospital simulation for real-world disaster response: design and validation of a virtual reality simulator for mass casualty incident management. J Trauma Acute Care Surg.

[16] Baetzner AS, Wespi R, Hill Y (2022). Preparing medical first responders for crises: a systematic literature review of disaster training programs and their effectiveness. Scand J Trauma Resusc Emerg Med.

[17] Vincent DS, Sherstyuk A, Burgess L, Connolly KK (2008). Teaching mass casualty triage skills using immersive three-dimensional virtual reality. Acad Emerg Med.

[18] McCoy CE, Alrabah R, Weichmann W (2019). Feasibility of telesimulation and Google Glass for mass casualty triage education and training. West J Emerg Med.

[19] Cone DC, Serra J, Kurland L (2011). Comparison of the SALT and smart triage systems using a virtual reality simulator with paramedic students. Eur J Emerg Med.

[20] Heuser J, Tolg B, Loer K (2023). Virtual staff teamwork during the pandemic - development of digital training formats for community emergency response during the coronavirus pandemic. [Online ahead of print]. Notf Rett Med.

[21] Luigi Ingrassia P, Ragazzoni L, Carenzo L, Colombo D, Ripoll Gallardo A, Della Corte F (2015). Virtual reality and live simulation: a comparison between two simulation tools for assessing mass casualty triage skills. Eur J Emerg Med.

[22] Lerner EB, Schwartz RB, McGovern JE (2015). Prehospital triage for mass casualties. Emergency Medical Services: Clinical Practice and Systems Oversight.

[23] Norman G (2010). Likert scales, levels of measurement and the "laws" of statistics. Adv Health Sci Educ Theory Pract.

